# Frequency of and Various Factors Associated with Stress, Anxiety, and Depression among Low Back Pain Patients

**DOI:** 10.7759/cureus.5701

**Published:** 2019-09-19

**Authors:** Syed Muhammad Azfar, Manal Abdulaziz Murad, Syeda R Azim, Mukhtiar Baig

**Affiliations:** 1 Orthopedics, Liaquat College of Medicine and Dentistry, Karachi, PAK; 2 Family Medicine, King Abdulaziz University, Jeddah, SAU; 3 Misc, Dow University, Karachi, PAK; 4 Misc, King Abdulaziz University, Jeddah, SAU

**Keywords:** lower back pain, mental distress, dass-21

## Abstract

Objective

To measure the frequency of depression, anxiety, and stress and its association with other variables i.e., age, gender, and off work hours among low back pain (LBP) patients attending an orthopedic outpatient department (OPD) at a private hospital in Jeddah, Saudi Arabia (SA).

Methodology

This is the cross-sectional study, which was done in a secondary care hospital of Jeddah, SA. Data was collected between the periods of 2017-2018. All patients who attended orthopaedic OPD with LBP were included in this study and were requested to fill the Depression, Anxiety, Stress Scale-21 (DASS-21) questionnaire. The gathered data were analyzed through the Statistical Package for Social Sciences (SPSS) version 23 (SPSS Inc., Chicago, IL). One-way analysis of variance (ANOVA) was used to compare the mean difference in depression, anxiety, and stress scores between genders, age, and number of leaves from their work.

Results

Three hundred sixty patients came to the orthopedic OPD with the primary complaint of LBP, 318 (88.3%) were male while 42 (11.7%) were female. The study showed that among these patients 24 (6.7%) subjects were suffering from the depression while 136 (37.8%) from anxiety and 167 (46.4%) from stress. Linear regression analysis showed that depression was negatively associated with age and stress was negatively associated with the off work because of the severity.

Conclusion

In conclusion, the findings of this study revealed that LBP and mental distress are related to each other. This finding urges physicians to check and treat the mental distress in patients with LBP for a better outcome.

## Introduction

Low back pain (LBP) is one of the most common diseases being experienced by about 70% of people during their lifetime. LBP is ranked as the number one cause of disability among 291 conditions, and sixth in terms of overall burden in the Global Burden of Disease 2010 study [1]. A recent study in Saudi Arabia (SA) found back pain top among ten frequent diagnoses in an orthopedic clinic [2]. Back pain not only affects the quality of life in patients but also imposes a high economic encumbrance on individual and society. Additionally, a plethora of evidence has demonstrated that back pain often co-occurs with psychological disturbance including depression, anxiety, and stress, which undesirably affects the outcomes of treatment [3]. Investigators also proved that improvement of the anxiety and depression status can help individuals control and manage pain in LBP [4-5]. 

In a review of sixteen research articles, Linton (2000) reported that in 14 studies revealed that, mental distress increased the risk for developing back pain [6]. There are several psychological theories about the development of psychological distress in chronic back pain. Some suggest that chronic back pain can lead to a diminished ability to engage in a variety of activities such as work, recreational pursuits, and interaction with family members and friends. This situation leads to a downward physical and emotional spiral that has been termed "physical and mental deconditioning" [7]. As the spiral continues, the person with chronic back pain feels more and more loss of control over his or her life. The individual ultimately feels totally controlled by the pain, leading to stress, anxiety or depression [7].

Another notion explains that pain and depression are closely correlated from the perspectives of both brain regions and the neurological function system, whereby chronic pain may lead to depression. One of the important causes of chronic pain leading to depression appears to be the common neuroplasticity changes in the occurrence and development of the two disorders [7]. 

The psychological aspects of the pain experience are often ignored, which can impede the recovery process. It is therefore timely to examine which psychological aspects should be addressed in consultations for back pain. In the past few decades, we have seen several research papers on the association of psychological issues to LBP. Many pieces of research have yielded consistent and convincing evidence of the possible relationship between back pain and mental health problems. However, the scarcity of epidemiological data from Asian nations has prevented doctors from a better understanding of how back pain and mental health are related? And how it will affect the course of treatments? Hence, the current study aims to measure the frequency of mental distress among the patients with LBP and its association with other variables i.e., age, gender and off work hours among patients attending an orthopedic outpatient department (OPD) at a private hospital in Jeddah, SA.

The results of our study may help health professionals to discover new and better treatment options.

## Materials and methods

This is the cross-sectional study which was done in a secondary care hospital of Jeddah, SA. Data was collected between the periods of 2017-2018. The approval for this study was granted from the hospital ethical committee and informed consent was obtained from all participants. All patients who attended orthopedic OPD with the presenting complaint of LBP were included in this study and they were requested to fill the Depression, Anxiety, Stress Scale-21 (DASS-21) questionnaire. It is an effective tool for clinical assessment of depression, anxiety, and stress. However, the assessment by this tool doesn't reflect the diagnosis. DASS-21 was preferred because it is a simple, reliable, and validated tool that can be used for research and clinical purposes [8-9]. The internal consistency of the DASS-21 questionnaire is reported high with Cronbach's α of 0.84 to 0.97 [10]. It is a 21-item shorter version of the DASS with three scales of seven items labeled: depression, anxiety, and stress. To complete the DASS-21, respondents are asked to circle a number 0, 1, 2, or 3 indicating how much the item applied to them over the past week, where 0 equals to “Did not apply to me at all,” 1 equals to “Applied to me to some degree, or some of the time,” 2 equals to “Applied to me to a considerable degree, or a good part of the time,” and 3 equals to “Applied to me very much, or most of the time" [10]. Demographic variables such as age, gender, and the number of leaves from work were also noted.

The gathered data were analyzed by the Statistical Package for Social Sciences (SPSS) version 23 (SPSS Inc., Chicago, IL). Descriptive-analytic methods were employed to analyze the gathered data. Frequencies and percentages were calculated for qualitative variables and mean ± standard deviation were calculated for quantitative variables. One-way analysis of variance (ANOVA) was used to compare the mean difference in depression, anxiety, and stress scores between genders, age, and number of leaves from their work. Linear regression was employed to explore the association of DASS-21 with age, gender, body mass index (BMI), off work due to the severity of pain, and job nature of patients of LBP. The P-value of less than 0.05 considered statistically significant.

## Results

Three hundred sixty patients came to the orthopedic OPD with the primary complaint of LBP, 318 (88.3%) were male while 42 (11.7%) were female (Table 1).

**Table 1:** Distribution of patients according to DASS (Depression, Anxiety, and Stress Scale) scores

DASS* Categories	Depression N(%)	Anxiety N(%)	Stress N(%)
No	336(93.3)	39(10.8)	193(53.6)
Mild	15(4.2)	66(18.3)	73(20.3)
Moderate	9(2.5)	25(6.9)	62(17.2)
Severe	0(0)	6(1.7)	27(7.5)
Extremely Severe	0(0)	39(10.8)	5(1.4)

The study showed that among these patients 24 (6.7%) subjects were suffering from the depression while 136 (37.8%) were suffering from anxiety, and 167 (46.4%) from stress (Table 2).

**Table 2:** Comparison of DASS-21 (Depression, Anxiety, and Stress Scale) score according to gender, different age groups, BMI, off work due to severity of pain, and nature of job BMI: body mass index

Variables	Depression Mean ± SD	Anxiety Mean ± SD	Stress Mean ± SD
Gender			
Male N=318	3.00 ± 3.60	7.62 ± 4.36	15.05 ± 6.51
Female N=42	3.01 ± 3.25	6.74 ± 4.94	15.47 ± 6.73
p-value	0.538	0.784	0.918
Age group			
<=30 N=76	2.76 ± 3.08	6.68 ± 4.61	14.63 ± 6.79
31-40 N=139	3.53 ± 3.88	7.51 ± 5.36	16.12 ± 7.03
41-50 N=74	3.16 ± 3.00	6.16 ± 4.18	16.46 ± 6.05
>50 N=71	2.11 ± 2.21	6.39 ± 4.76	13.83 ± 6.29
p-value	0.025	0.193	0.039
BMI groups			
Underweight N=0	-	-	-
Normal N=36	2.00 ± 1.85	5.56 ± 3.25	14.11 ± 6.23
Overweight N=42	2.90 ± 2.62	7.48 ± 4.90	17.52 ± 7.31
Obese N=282	3.16 ± 3.50	6.91 ± 5.03	15.28 ± 6.61
p-value	0.025	0.193	0.039
Off work due to severity of pain			
Absent < 4 days N=141	2.89 ± 2.35	6.65 ± 4.29	16.31 ± 6.50
Absent > 4days N=18	2.67 ± 2.66	5.22 ± 4.12	15.44 ± 6.78
No Absence N=144	3.28 ± 4.00	6.96 ± 5.27	14.94 ± 6.51
No disability N=53	2.83 ± 3.63	7.77 ± 5.43	14.83 ± 7.33
No pain N=4	1.50 ± 1.00	4.00 ± 3.27	9.00 ± 8.25
p-value	0.678	0.234	0.119
Nature of job			
Desk N=53	3.06 ± 3.81	6.04 ± 4.32	14.72 ± 7.10
Housewife N=42	2.86 ± 3.16	7.57 ± 4.20	14.14 ± 5.66
Manual N=118	2.83 ± 2.82	6.59 ± 5.08	15.22± 7.22
Non Manual N=147	3.18 ± 3.49	7.12± 5.06	16.20 ± 6.33
p-value	.836	.370	.238

Linear regression analysis shows that depression was negatively associated with age and stress was negatively associated with the off work because of the severity (Table 3). The people between 31-40 and 41-50 years were more depressed and stressed as compared to other age groups. Overweight and obese were having more stressed and depressed as compared normal weight (Table 3). No significant difference of the scores of DASS-21 was found with a number of leaves to the severity of pain, between genders and nature of the job.

**Table 3:** Linear Regression showing association of DASS-21 (Depression, Anxiety, and Stress Scale) with age, gender, BMI, off work due to severity of pain, and nature of job among patients of low back pain BMI: body mass index

Model	Depression		Anxiety		Stress	
	Beta Coefficients	p-value	Beta Coefficients	p-value	Beta Coefficients	p-value
(Constant)	1.924	.361	3.923	.200	19.454	.000
Age	-.031	.045	-.029	.198	-.035	.275
Gender	.123	.847	-.799	.389	-.483	.715
BMI	.053	.162	.103	.063	.002	.981
Off work severity	-.041	.788	.058	.794	-.712	.026
Job Nature	.220	.357	.472	.174	-.104	.833

## Discussion

As discussed above, back pain is one of the leading causes of disability across the globe and there is also a wealth of evidence that it negatively impacts the quality of life and heightens the risk of mental health problems and mood disorders [1-2]. The present study also found that a significant number of patients with LBP had mental distress (see Table 2). Among 360 patients of LBP, 6.7% of patients had depression, 37.7% had anxiety, and 46.4% had stress. These findings are parallels to the findings of another current study, which showed higher mental distress among LBP patients as compared to the control group [11]. This might be because anxiety and stress can actually increase the perception of pain and reduce pain coping skills [12].

Moreover, it is also observed that a treatment that targets both of mental distress and lower back pain simultaneously seems to have better results [12]. A recently published study found that an intervention consisting of physiotherapy combined with cognitive functional therapy was superior to a treatment option consisting of physiotherapy focusing only on the physical aspects of pain and disability [13]. Due to prodigious evidence in favor of the parallel relation of chronic pain and mental health Bruns & Disorbio (2014) proposed that the psychological evaluation should be an integral part of the diagnostic workup for chronic pain [14].

In this study, we found that out of 360 patients with lower back pain, 88.3% are males, while 11.3% are females. This gender and age distribution of patient reflects the fact that the population of Jeddah comprises a large number of expatriates and many of them are male workers who are living alone without family. Although there is a significant difference in gender distribution, there was no significant difference in DASS21 was found among male and female subjects. However, research shows that female subjects are more prone to mental distress as compared to their male counterparts [15].

In terms of severity, we have found in our study that patients with LBP have more moderate and severe stress and anxiety as compared to the depression in this category there is no patient had moderate or severe depression. This finding might suggest that back pain may cause stress and anxiety, which if not treated, may progress to depression. A scientific review paper claims that chronic stress can lead to the structural degeneration and impaired functioning of the hippocampus and the prefrontal cortex, which might increase the risk of developing neuropsychiatric disorders, including depression and dementia [16].

In our study, we found that patients between 31-40 and 41-50 years were more depressed and stressed as compared to other age groups. Here is an interesting finding in our study, which shows that depression was negatively associated with age. Another study by Jorm et al. revealed that depression and anxiety decline as age increases, which, they argue, is due to a significant reduction in exposure to a number of risk factors, the most important being work-related stressors [17]. Our study shows that work-related stressors reduce significantly as age increases. The majority of patients in the older age groups are retired, and being unemployed due to pain, a significant work-related stressor becomes much less prevalent as age increases, which may partially account for lower DASS-21 scores in the older age groups. Another potential reason for age differences is that “aging of the brain may affect emotional responsiveness [18]. Research has shown that older adults are less likely to attend to and remember negative compared with positive emotional material. Another possibility may relate to the acceptance of pain [18]. It is conceivable that older people in pain may be more accepting of their pain as something to be expected after 60 or more years of active life. Conversely, persisting pain may be less accepted by the young adult group as displayed in our study [19].

Another significant finding in this study is that stress was negatively associated with the number of absence from work. While stress is negatively associated with the number leaves, this means that stress improved with taking off from the work. American Psychological Association (APA) has found work, as a significant source of stress, and people can't always avoid the tensions that occur on the job even if they love their job [20]. We also found in this study that overweight people were more stressed and depressed as compared to normal weight. Jorm et al. (2003) also found that obesity has an association with anxiety, depression, and lower wellbeing [17]. There is evidence that obesity and depression are the established risk factor for back pain and can be occurred combined. It is also suggested that LBP could also cause less physical activity and thereby result in obesity and psychological ill health [21]. 

Limitations

Generalization of the finding of this study has to be considered with caution because we did convenience sampling. However, future researches may investigate different populations, taken into consideration the previous issues in order to have a stronger argument for extrapolation of the results.

## Conclusions

In conclusion, the findings of this study revealed that LBP and psychological issues are related to each other. A notable number of patients had anxiety and stress, and a small percentage of patients were depressed. Moreover, depression was negatively associated with age, stress, and was negatively associated with the leaves from work. Therefore these findings acknowledge the importance of psychological assessment and treatment, in addition to biomedical aspects when managing patients with LBP. This study divulges that clinicians should be aware of potentially high rates of emotional distress syndromes in chronic low-back pain and enlist mental health professionals to help maximize treatment outcomes. Further research is required to find out more about the links between LBP and mental distress to ensure the development of effective treatments.
